# Scenarios of land use and land cover change in the Colombian Amazon to evaluate alternative post-conflict pathways

**DOI:** 10.1038/s41598-023-29243-2

**Published:** 2023-02-07

**Authors:** William-J. Agudelo-Hz, Natalia-C. Castillo-Barrera, Murcia-García Uriel

**Affiliations:** GIS and RS Laboratory, Functioning Models and Sustainability Program, Amazon Institute for Scientific Research SINCHI, Sede de Enlace Bogotá, Calle 20 # 5-44, Bogotá, Colombia

**Keywords:** Ecology, Conservation biology, Ecological modelling, Environmental impact

## Abstract

Pastures and crops have been expanding at an accelerated rate in the forests of the Colombian Amazon since the peace accords were signed in 2016. The rapid loss of tropical rainforests is threatening the integrity of protected areas and connectivity in the Amazon and other natural regions. In the context of the post-conflict stage, a set of land use and land cover change scenarios were constructed for the Colombian Amazon for the year 2040, using expert coherent narratives. Three scenarios were designed: trend, extractivist, and sustainable development. Historic land use change and driving factors were analyzed throughout 14 transitions between the years 2002 and 2016, based on the interpretation of Landsat images and their relationship with 29 driving factors using artificial neural networks. The Markov chain model was calculated for the transitions, and the change allocation model was parameterized to spatially simulate the scenarios. The results showed that the LULC model calibration and validation were satisfactory (0.91). The sustainable development scenario that considers strong policies for the conservation of forests and implementation of sustainable production projects was the option with greater values for conserved forests and secondary vegetation in recovery, adding ~ 42 million hectares by 2040. The other scenarios showed that the Colombian Amazon will lose ~ 2 million hectares of forests in the trend scenario and ~ 4.3 million hectares in the extractivist scenario, based on the reference year (2016). In the trend scenario, pastures and crops could increase by 48%; and, in the extractivist scenario, these would increase by 117%, changing from ~ 3.9 to ~ 8.6 million hectares. We hope that the scientific contribution of this study will be relevant for informed discussion in decision-making and provide a framework for building a peaceful territory.

## Introduction

In Colombia, after a long history of civil war, the current conservation of intact forests is threatened by accelerated deforestation^1,2^. In forested regions such as the Colombian Amazon, the historical armed presence of the Revolutionary Armed forces of Colombia (FARC-EP) limited deforestation, restricting the expansion of illegal activities such as coca crops and illegal cattle ranching, as well as the advance of commercial and subsistence crops^3^. After the peace accords in 2016 between the government of Colombia and FARC-EP, the dynamics of the occupation in this territory, which were hidden during the conflict, became more evident, accelerating changes in natural covers and land uses^3^. In the Amazon, activities such as illegal mining, timber extraction and land grabbing play an important role in the illegal economy in the territory, increasing speculation about the value of cleared lands in public zones and protected areas^4,5^.

Data derived from satellite monitoring of the coverage of the Colombian Amazon show that, from 2017 to 2020^6^, the loss of forests and the frequency of forest fires increased, even in protected areas, giving way to extensive areas with pastures, secondary vegetation, and fragmented forests^1,2,7^, putting the regional connectivity of the Amazon with the Andean mountain system and the Orinoquía region at risk^2^.

This post-conflict period provides prospective tools for local decision-makers for the future of the territory so they can prioritize the following: (i) strategies in the territory that prevent deforestation, (ii) control of illegal expansion of pastures, (iii) establishment of productive alternatives and sustainable forest management, and (iv) productive restoration in agricultural landscapes. This would facilitate reconciliation of economic growth and local livelihoods with conservation of the environment and provide ecosystem services of vital importance for life on the planet through lasting social agreements^9,10^. Therefore, evaluating the effects of recent changes and development trends and alternatives under different scenarios of land use changes in the context of a post-conflict stage is important for making informed decisions^11,12^.

Scenarios of land use and land cover (LULC) change have been used as tools to support territorial planning decisions in regions of large tropical forests with the purpose of protecting biodiversity and reducing emissions from deforestation and degradation^12–15^. LULC change scenarios are useful for visualizing and analyzing the effect of different developmental pathways, helping decision-makers to formulate scientific strategies and implement plausible policies that conserve natural ecosystems and provide ecosystem services^16–19^. For instance, a LULC scenario that jointly assessed climate change, elaborated for the Amazon basin, indicates that, if 40% of the rainforest is lost, the forests could head to a tipping point or point of no return, where the remaining forest would eventually transform into a savanna ecosystem^20^. This have inferred that the loss of Amazon forests could lead to a reduction in rainfall, creating favorable conditions to potentially alter forest structure and completely modify the Amazon biome^20–22^.

In the context of environmental management, scenario-based decision making is part of a work proposal called strategic foresight or scenario planning^23–26^. The goal of strategic foresight is to explore possible futures, its consequences on decision making and actions that promote more desirable futures, thus shifting the focus from forecasting a single future to exploring multiple alternative futures in systems of high uncertainty^23,26–28^.

One of the approaches for the construction of scenarios is the so-called “Story and Simulation” (SAS)^29,30^, in which scenarios are elaborated from plot stories called narratives that contain the hypotheses that underlie potential changes in the future. The analysis of these narratives makes it possible to translate a qualitative model into a quantitative model and subsequently create spatially explicit simulations of future land uses. The purpose of this study was to analyze the driving factors that influence land use and land cover change patterns in the Colombian Amazon forest and to simulate a set of plausible future scenarios up to the year 2040, in order to guide adequate sustainable decision making in the region.

The study area was the Amazon region of Colombia, an area that represents 7.9% of the Greater South American Amazon^31^ and has experienced accelerated deforestation rates in the last 5 years^32^. In this study, changes in land cover and land use and transitions between different vegetation cover (e.g., amazon forest to pastures and crops) were analyzed using two maps obtained from the interpretation of Landsat TM+, OLI images by the SINCHI Institute for the years 2002 and 2016, at a scale of 1:100,000^33^ and resampled at a pixel size of 60 m. The probability of change was calculated for the 14 most relevant transitions between coverages, using artificial neural networks-multilayer perceptron (ANN-MLP) in TerrSet^34^, using 29 driving factors associated with the changes. The hypotheses of future conditions for each scenario in the narratives were translated into quantitative parameters to elaborate the maps of future LULC changes, including modifications in the Markov chain probability matrix and incentives and spatial constraints within the model to produce the space simulations up to 2040.

This research showed novel advances in the following aspects: (1) different views of experts for the development of a new set of contrasting scenarios for land use changes in the Colombian Amazon, unlike previous studies that developed simulations of changes in land covers based on global developmental hypotheses or policies based on the country's environmental regulations^35^; (2) the set of variables, the selected spatial resolution, and the ANN-MLP approach satisfactorily explained the factors of transformation and produced a model with a high predictive capacity for changes in the territory in the different transitions; (3) the simulated maps of the future until 2040 showed the potential effects of different socioeconomic development pathways in the post-conflict stage, providing the most sustainable development for the conservation of forests and ecosystem services in the Colombian Amazon. The scientific contribution of this study will be relevant for informed discussions in decision-making, providing a framework for thinking about building a peaceful and prosperous region, with conservation of intact forests in one of the most important areas of Colombia for biodiversity, water production, and carbon sequestration on a global scale.

## Methods

### Study area

**Figure 1:**
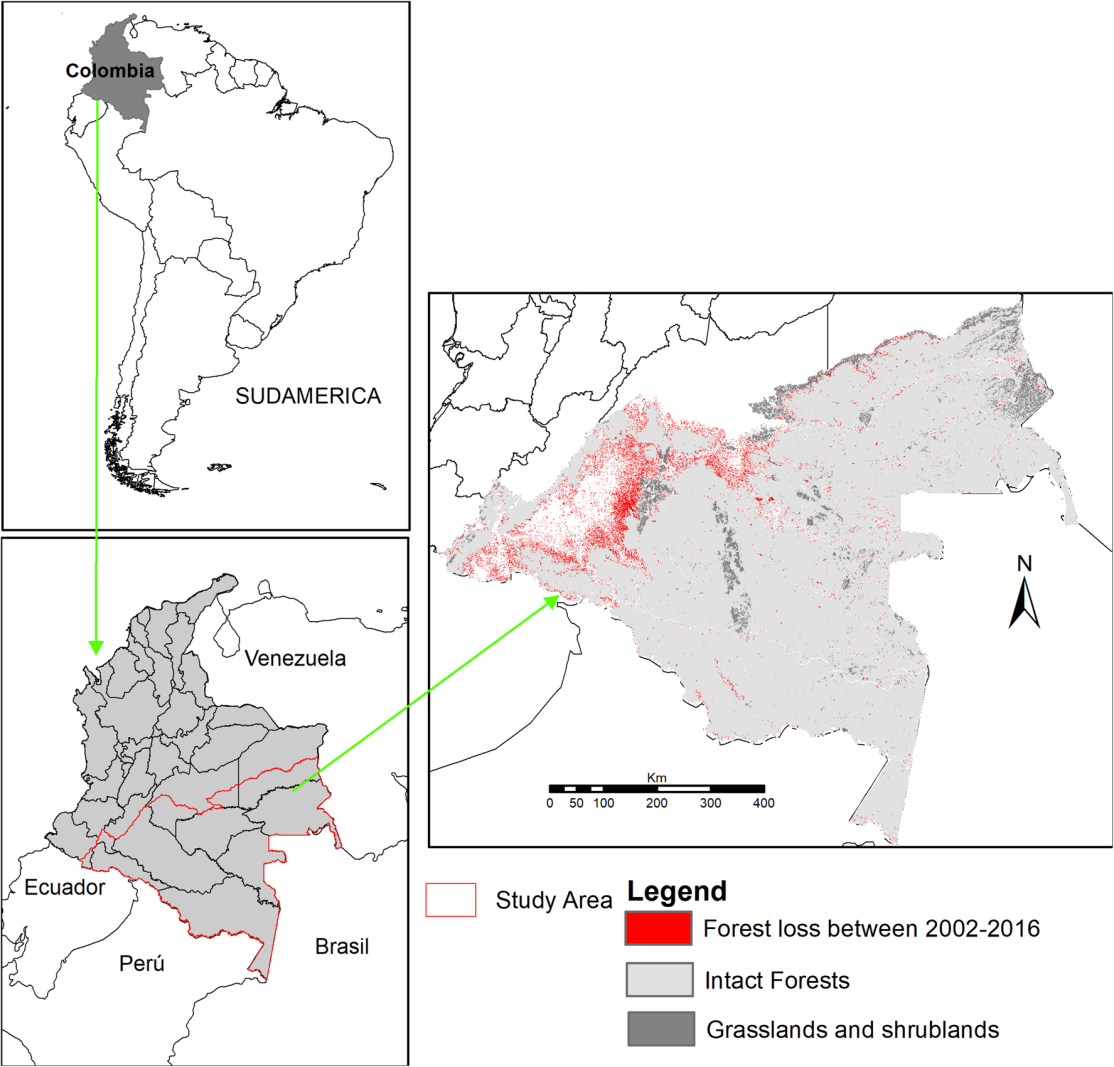
Study area. Colombian Amazon and location of Amazonian tropical forests that were lost between 2002 and 2016. (Maps were generated using software ArcGis 10.7.1 https://www.esri.com).

In Colombia, the Amazon region represents 42.3% of the territory with an estimated area of 483,164 km^2^. In this area, 14% is dominated by agricultural lands, secondary vegetation and fragmented forests. Currently, 86% of the area corresponds to natural areas in a good state of conservation, where forests are the dominant coverage^6^. In the northwest area, the region borders the Andean Cordillera and Orinoquía to the north. The political-administrative division includes the departments Amazonas, Caquetá, Guainía, Guaviare, Putumayo and Vaupés, and part of the departments Cauca, Meta, Nariño and Vichada. The human population is estimated at ~ 1.4 million, with a density of 2.5 inhab/km^2^. Internal conflict and poverty make this region one of the most important population dynamics in the country in terms of displacement^36^. The geographical location of the study area and the spatial pattern of the loss of forests that occurred between 2002 and 2016 are shown in Fig. 1.

### Land cover maps and variables for change analysis

Thematic land cover maps used in this research were produced by the Colombian Amazon Land Cover Monitoring System (SIMCOBA) of the Amazon Institute for Scientific Research SINCHI (https://siatac.co/simcoba/). SIMCOBA has prepared land cover maps for the periods 2002, 2007, 2012, 2014, 2016 and 2018. Three of the land cover maps prepared were used in this study: 2002, 2016 and 2018 a scale of 1:100,000^33^. The maps were generated from the visual interpretation of a mosaic of Landsat 5 Thematic Mapper (TM) and Landsat 8 Operational Land Imager (OLI) images, using the PIAO technique (Photo Interprétation Assistée par Ordinateur). The classification categories of the land cover maps were based on the Corine land cover methodology adapted for Colombia^37^.

The SIMCOBA system calculates the annual rates of Amazon forest loss (forest loss/ha/annual) by comparing the cover maps of the last two periods and subtracting from the previous map those forests that are no longer present in the most current map (Fig. 3). This process only considers the forests loss and the permanent forests. New forests due to natural regeneration or restoration are omitted in the calculations^6^.

To facilitate the interpretation of changes and cover transitions, the classification categories of the maps were re-categorized into 7 types: "Amazon forests", "floodplain forests", "fragmented forests and secondary vegetation", "grasslands and shrublands", "water bodies and wetlands", "pastures and crops" and "urban and artificialized cover". The land cover maps were resampled at a resolution of 60 m × 60 m to facilitate the computational analysis of the explanatory model, the simulations of the scenarios, and to keep the detailed spatial resolution of the coverage and explanatory variables^16^.

A geospatial database was created with a set of variables for the cover changes to create an explanatory model for each transition. Driving factors of change are grouped into the following variables: (1) accessibility, (2) climate, (3) landscape features, (4) production practices and environmental degradation, (5) landscape management, (6) socioeconomy, and (7) soil characteristics. We considered 41 explanatory variables (see supplementary information Table S1).

Accessibility variables such as roads and navigable rivers were obtained from the geodatabase at a scale of 1:100,000 of the Agustín Codazzi Geographical Institute of Colombia (IGAC). Bioclimatic temperature data were obtained from Worldclim v1.4^38^. Cover variables (e.g., patch sizes Amazon forests and distance to pastures and crops) were created using the software ArcGis (v.10.7.1)^39^ from the 2002 land cover map to understand which drivers were more influential in the dynamics of land-use changes since 2002 that resulted in the distribution of land cover in 2016.

Degradation variables, such as advances of the agricultural frontier, were obtained from the Territorial Environmental Information System of the Colombian Amazon (SIAT-AC)^40^; livestock density data came from the Colombian Agricultural Institute (ICA); the fire density were processed from MODIS and VIIRS images (https://siatac.co/puntos-de-calor/); and the location of mining titles was obtained from the National Mining Agency.

The information on the landscape features and socioeconomic variables was obtained from different sources: (1) the limit of the protected natural areas was provided by the National System of Protected Areas (SINAP)^41^, (2) the Amazon Forest Reserve areas (Second Law of 1959) were obtained from the Ministry of Environment and Sustainable Development (MADS), (3) the location of the indigenous reservations was provided by the Ministry of the Interior, and (4) the limits of the areas of Indigenous Reservations and the legal status of the Amazonian region were obtained from the SINCHI cartographic database^40^.

Socioeconomic information was spatialized from data from the National Administrative Department of Statistics (DANE). Soil-type data were obtained from IGAG, and topographic and altitudinal variables were derived from a DEM at 100 m resolution from the Advanced Spaceborne Thermal Emission and Reflection Radiometer (ASTER V003) sensor^42^. All explanatory variables were resampled at a resolution of 60 m.

### Patterns of land cover changes and transitions

**Figure 2:**
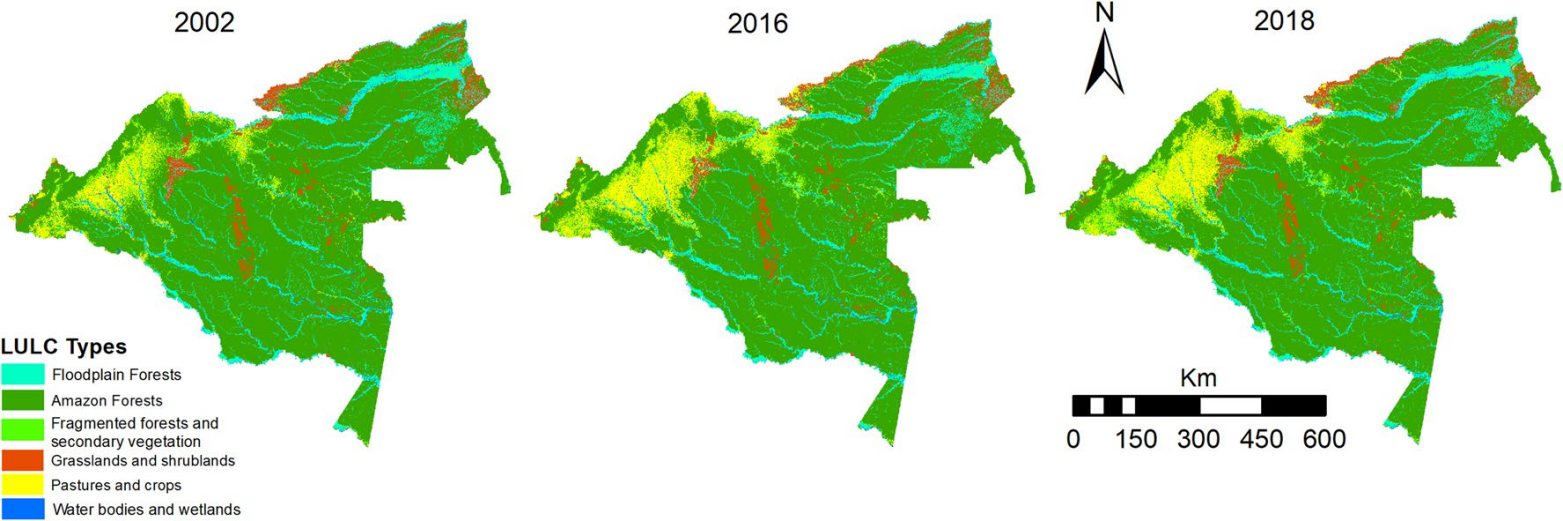
Land cover maps 2002, 2016 and 2018, produced by the Colombian Amazon Land Cover Monitoring System (SIMCOBA) of the Amazonian Research Institute SINCHI (Source: Open Data—SINCHI Institute https://datos.siatac.co/pages/coberturas) (Maps were generated using software ArcGis 10.7.1 ).

The transformation patterns of territory are mainly defined by human intentions and the activities that these groups plan to develop after making the land cover changes, as well as the dynamics of vegetation regeneration^43^. In this study, these changes in the study area were obtained and analyzed employing the Land Change Modeller (LCM) module of TerrSet^34^ and using the land cover maps for 2002 and 2016 as input information (Fig. 2).

To represent dynamics and changes in the vegetation during the study period, a total of 14 transitions of greater importance in terms of area were considered (transitions with an area < 5000 ha were ignored) to reduce the complexity in the analysis of land use changes from the multiple possibilities of transitions that can be configured (Table 1). Three submodels of changes that grouped transitions were defined^8^: (1) submodel of degradation was defined by the changes that "Amazon forests" and "floodplain forests" may experience towards "fragmented forests and secondary vegetation " and “grassland and shrubland”, either from selective logging, fire or small-scale activities such as the establishment of crops or pastures amid continuous blocks of intact forests; (2) Submodel of substitution included the transition from "Amazon forests", "floodplain forests", and "fragmented forests and secondary vegetation" to "pastures and crops". These transitions are usually illegal as the replacement of Amazonian forests to extensive areas of pasture for livestock takes place mainly on lands that belong to the Colombian state, with no authorization of the country's environmental authorities. (3) The regeneration submodel grouped the transitions that show recovery from a degraded cover to a forest cover or areas with abandoned “pastures and crops” towards “fragmented forests and secondary vegetation” in recovery.

**Table 1:** Patterns of landscape transformation and Land-use and cover transitions between 2002 and 2016.

Patterns of landscape transformation	Land-use and cover transitions	Area (ha)
Degradation	Floodplain forests to grasslands and shrublands	8909
	Floodplain forests to fragmented Forests and secondary vegetation	48,740
	Amazon forests to grasslands and shrublands	8935
	Amazon forests to fragmented forests and secondary vegetation	664,181
Substitution	Grasslands and shrublands to pastures and crops	164,121
	Water bodies and wetlands to pastures and crops	33,356
	Floodplain forests to pastures and crops	45,562
	Fragmented forests and secondary vegetation to pastures and crops	681,206
	Amazon forests to pastures and crops	923,154
Regeneration	Grasslands and shrublands to fragmented forests and secondary vegetation	23,678
	Pastures and crops to grasslands and shrublands	15,927
	Pastures and crops to fragmented forests and secondary vegetation	385,628
	Fragmented forests and secondary vegetation to floodplain forests	13,706
	Fragmented forests and secondary vegetation to Amazon forests	75,832

The results showed that the substitution submodel was the most important pattern of transformation in the Colombian Amazon from 2002 to 2016, because the transition to “pastures and crops” was ~ 1.85 million hectares (Table 1). The submodel of degradation was dominated by the transition from "Amazon forests" and "floodplain forests" to "fragmented forests and secondary vegetation" with ~ 0.66 million hectares. In the submodel of regeneration, the most significant process occurred with "pastures and crops" where 0.38 million hectares transitioned to "fragmented forests and secondary vegetation", giving rise to important areas of vegetation in recovery.

### Neural network analysis and land use change factors

The artificial neural network multi-layer perceptron (ANN-MLP) is a multivariate statistical algorithm of machine learning widely used in the analysis of factors associated with changes in land use^44–46^. MLP algorithm used in the LCM is an adaptation specially designed for the land change analysis^34^. ANN-MLP is a suitable method of classification to solve non-linear relationships in complex data sets^47–51^ such as those in this study. ANN-MLP is made up of the elements shown in Eq. (1):

1 $$y=f\left(z\right) \mathrm{and} z={\sum }_{i=0}^{n}{w}_{i}{x}_{i},$$

where, ${x}_{i}$ are the input values or training data of the variables; ${w}_{i}$ are the weights of the respective variables in the neural network; and *n* is the number of variables^52^. To solve the relationship between the explanatory variables and the response variable, the network adds one or more hidden layers of neurons connected by nodes (multi-layer) to find the most appropriate solution in the model (learning phase). The performance of the algorithm is controlled by two training parameters that are key for the application of an ANN-MLP (the learning rate and the “momentum”) because they control the speed and efficiency of the learning process^49^. The values of $z$ are multiplied by the transfer function or sigmoidal activation function $f$, whose output values, $y$, are the probability of change for each transition^51^. Under this criterion, the relationship of the factors for land use change for the 14 transitions were analyzed using ANN-MLP in TerrSet^34^, starting with a reduced set of driving factors. To avoid including slightly relevant information during the learning phase of the neural network, Cramer's V coefficient test was performed, which indicates the degree of association of each driving factor with the distribution of the land cover categories, that is, variables with V ≥ 0.15^34,53^. Finally, it is necessary to declare whether the variables are dynamic or static. This is important for the projection of cover simulations, because dynamic variables are responsible for change over time, such as proximity to roads or previously deforested areas; and they can be recalculated at regular intervals to update the progress of transitions over time during the course of a simulation^52^.

The results were evaluated using two precision statistics: the “accuracy rate” (AR) and the “skill measure” (SM), using a subset of validation data (50% training/50% testing). The “accuracy rate” evaluates the ability of the algorithm to predict the correct classes of validation pixels after each iteration to train the network^34^. AR values greater than 70% indicate that the submodel has good explanatory power. The "skill measure" indicates whether the prediction results are better than chance. SM values vary from + 1 (perfect prediction) to − 1 (worse than chance); a value of 0 indicates that the results are not better than chance^34^. The analyses were developed in the submodule “transition potentials” of the Land Change Modeller (LCM) of TerrSet. For each transition, a transition potential map was produced, whose pixels reported continuous values from 0 (no probability of change) to 1 (high probability of change) and evaluation metrics.

### Scenarios narratives and parameterization of simulations

Since the Peace Agreement, pastures and crops have been expanding at an accelerated rate in the forests of the study area^10^. The rapid loss of tropical rainforests is threatening the integrity of protected areas and connectivity in the Amazon and in other natural regions^8^. Based on this framework, LULC change scenarios have been developed to explore the effect of different biophysical and socioeconomic factors on the future of land use^54–56^ in the Colombian Amazon for the year 2040. For this purpose, three storylines were prepared by a group of 6 experts with wide environmental and scientific knowledge of the region, using interviews^30^. Expert interviews were orientated to briefly narrate events and contextualize factors that should be present and will drive the future of the Amazon in the next 20 years. Based on the interview results, three narratives were defined to build three scenarios: the trend scenario (BAU), the extractivist scenario, and the sustainable development scenario (see Table 2).

**Table 2:** Alternative scenarios and brief narratives developed with participatory interviews.

	Tools for land use planning: scenarios of LULCC
Alternative scenarios	Narrative
Trend	Bussines as usual (BAU)
	The dynamics of transformation of natural covers are maintained. Government interventions are not enough to stop deforestation and fires. Extensive cattle ranching and land grabbing continue to be the main drivers of transformation of primary and secondary forests to grasslands; however, initiatives such as conservation agreements promote landscape-scale improvements. The commitments of the peace agreement regarding land restitution and replacement of illicit crops were not consolidated
Sustainable development	Forests and peace
	Policies are put in place to completely stop deforestation and control forest fires. Property titles are not granted for deforested lands. Cattle ranching is reduced. Conservation agreements transform deforested areas into resilient landscapes through agroforestry, fish farming and silvopastoral systems. Secondary vegetation is used for forest restoration to accumulate carbon, biodiversity and obtain goods and services. The governance of the territory has solid citizen participation. Agreements on land redistribution and support for the substitution of illicit crops are implemented. Violence in the territory is reduced
Extractivist	Violence, livestock, and grains
	There is no control over deforestation and fires. An increase in demand and prices for grains and meat causes large areas of untouched forests to be illegally converted to cattle ranching and growing agribusiness of soybean, corn, and oil palm crops. Deforestation increases to 40% of the trend rate. Extractive activities such as mining, and logging are strengthened in the territory. New settlements are created in jungle areas, strengthening road infrastructure. Violence impacts the community leadership that demands fairer conditions for the conservation and development of the territory

In the trend scenario simulation, the Markov chain model in TerrSet^34^ was used to obtain the probability matrix, and it was projected to 2040. A Markov chain model is a random stochastic process that calculates the probability of land cover permanence and transition based on the analysis of historical changes, providing a framework for analyzing future land use demand^57^. Markov probability matrix have been widely used for the analysis and modeling of changes in LULC^16^. Such a matrix shows the probability of a land use/cover change from one state to another taking place within a specified time period^58^. An advantage of the Markov model is that the transition probability matrix can be modified by taking account of future land-use demands, based on current or future land-use management policies or on the narratives of local experts knowledgeable about environmental issues.

**Figure 3:**
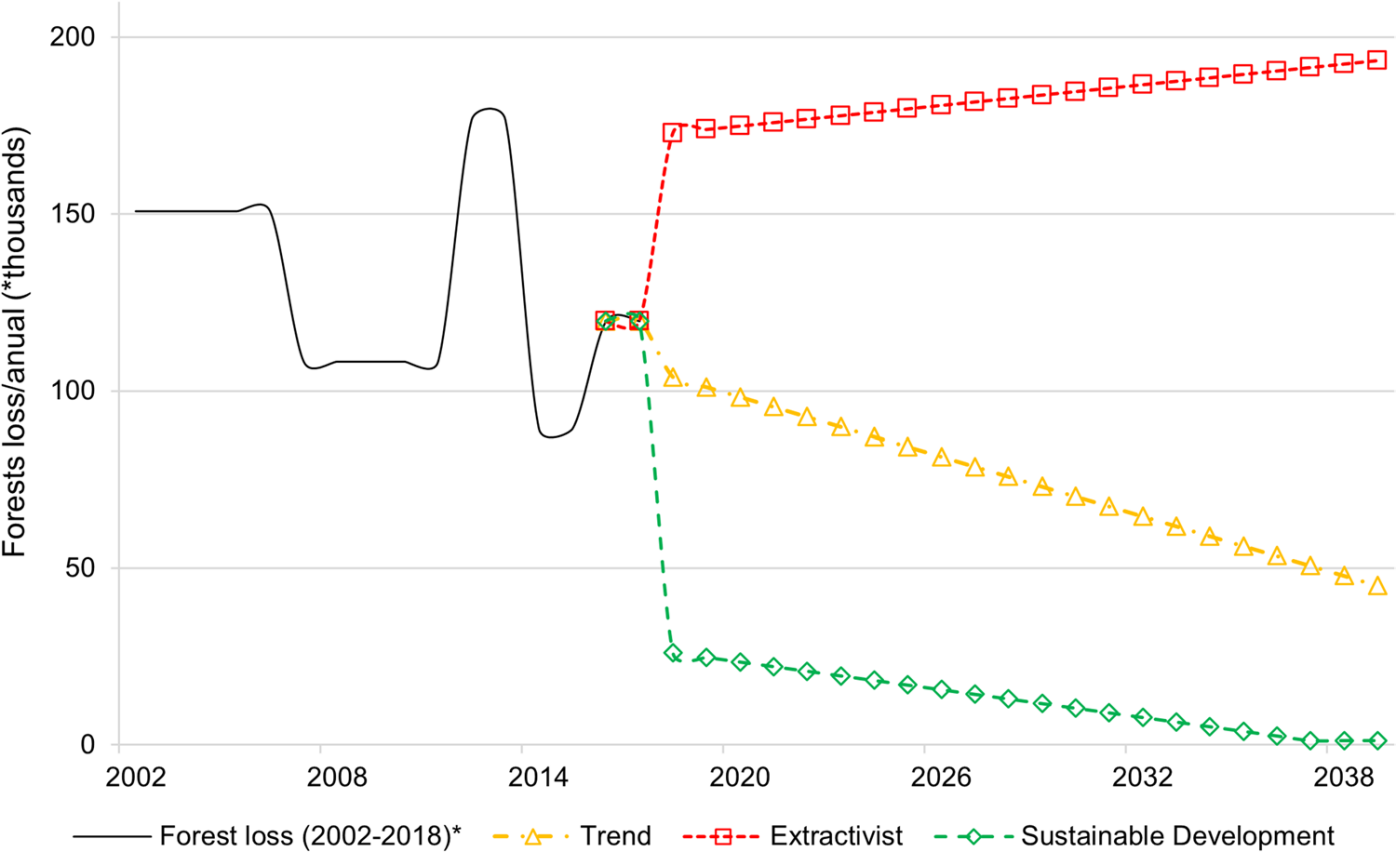
Projection of the annual forest loss until 2040. Trend scenario: based on losses observed between 2002 and 2016. Extractivist scenario: based on losses observed between 2008 and 2016 plus an annual increase of 40%. Sustainable development scenario: based on an 80% reduction in average forest loss (2002–2016) to a 99% reduction in 2040. *The average annual rates of forest loss between 2002 and 2018 based on SIMCOBA data (Source: https://siatac.co/coberturas-100k/).

**Figure 4:**
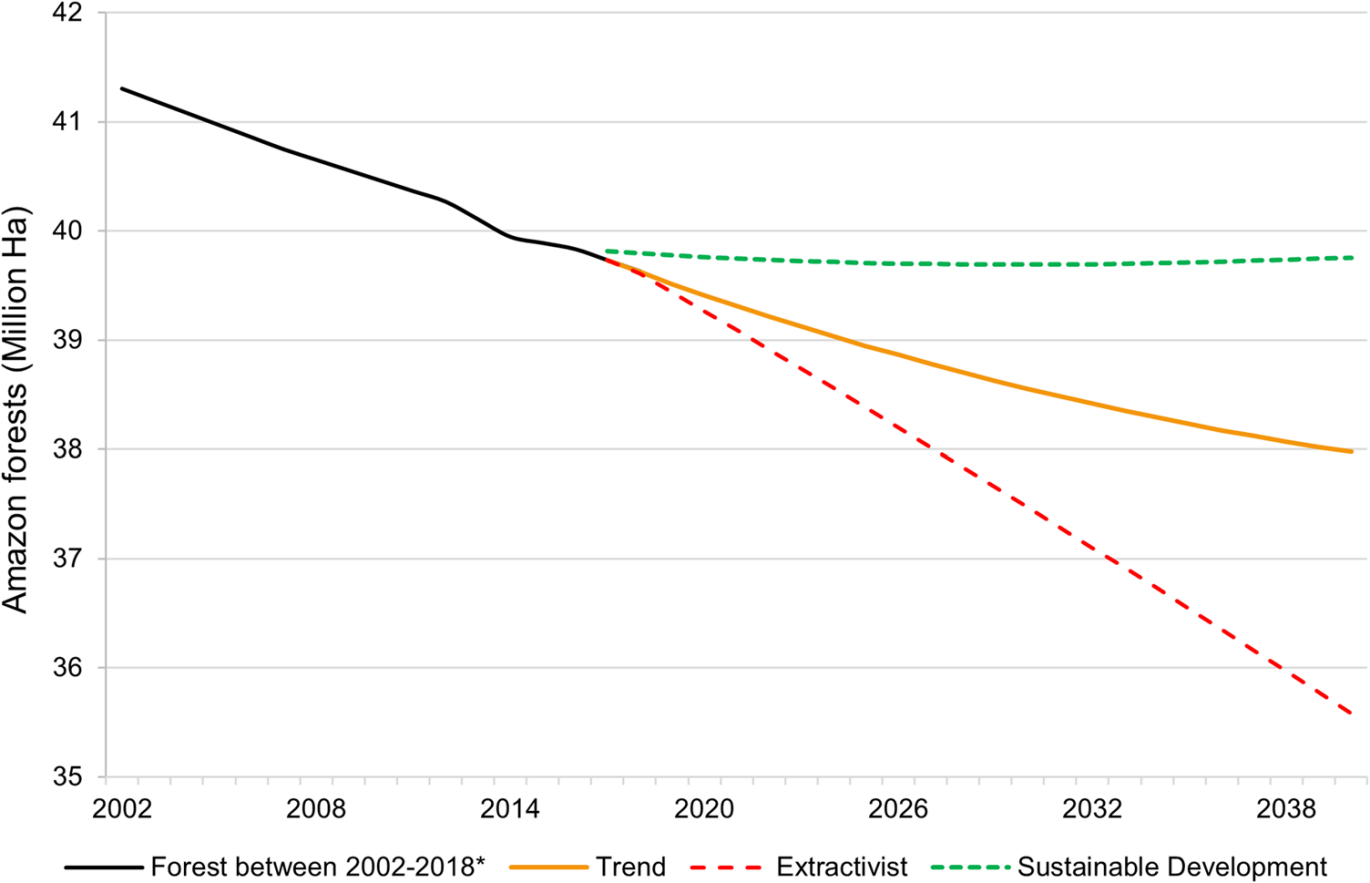
Projection of the area of Amazon Forests to 2040 based on projected annual forest loss for each scenario. *The area of Amazon Forest between 2002 and 2018 calculated based on SIMCOBA data (Source: https://siatac.co/coberturas-100k/).

Two external models of probability of change were created from the Markov chain matrix of the trend scenarios: one for the extractivist scenario and one for the sustainable development scenario. The probability potential change values were repeatedly modified to accomplish the projected land use demands in each scenario. Based on the annual rates of Amazon forest loss calculated between the years 2002 and 2016, an average was estimated, and this value was used to calculate the increase and reduction in forest loss in the extractivist scenario and in the sustainable development scenario (Figs. 3, 4).

Because the narrative adds importance to the current conservation agreements that have been developed in the study area^59^, a map was generated for rural associations that have a conservation and production program as an incentive in the transition from "pastures and crops" to "fragmented forests and secondary vegetation", using this transition as an approximation of the effect on the recovery of vegetation in these areas. The parameterization in the extractivist scenario simulation included a year-to-year increase in the rate of forest loss up to 40% to 2040 as compared to the average rate between 2008 and 2016, and it included a delimitation of areas for mining titles as an incentive for the potential loss of Amazonian forests that may occur in this scenario. The scenario for sustainable development involved an immediate reduction in forest loss (− 80% of the average annual rate) and a gradual reduction year by year to − 99% to the year 2040, as compared to the average rate between 2002 and 2016. A total restriction of the loss of forests in protected areas, indigenous reservations, and on land slopes greater than 100% was also applied. Similarly, sustainable development actions would impact productive reforestation in prioritized restoration areas as well as properties that make up rural associations where conservation agreements are developed.

### Simulation in LCM and validation

The ANN-MLP analysis evaluates the effect of different drivers on land cover change over a historical period, and it generates a map of suitability or probability of change that indicates where change will potentially occur. The LUCC model operates under the assumption that these drivers will continue to act in the future^60^. The Markov chain model, on the other hand, calculates the amount of change based on historical land use change data or an external model that reflects expectations under a particular scenario. Therefore, before projecting the simulation of scenarios, it is necessary to evaluate the precision of the model in order to simulate changes in land/use cover^51^. A spatial simulation was projected for 2018 and was compared with the land cover map for 2018 made by SINCHI Institute.

The precision of the model was evaluated based on two indices: (i) the general kappa index (K)^51,52^ and (ii) the k index of agreement (KIA)^61^. These two indices are useful to evaluate the following: (1) the ability of the explanatory model (ANN-MLP) to predict the location of the changes and (2) the projection of the amounts of change of each land cover by Markov chain analysis.

The K index assesses the concordance of the simulation in general for all covers with respect to the real data provided by the land cover map for 2018, expressed in values of 0–1; a kappa greater than 0.80 is a reasonable level of agreement. The KIA for land cover is a statistical measure of the difference between an observed agreement between two classifications versus agreement by chance^61^. KIA goes from 0–1; values of 0 mean no better than chance, and 1 means a perfect match.

## Results

### Selection of driving factors

The Cramer’s V test evaluated the influence of each of the 41 driving factors on the distribution of each category of land use in the 2016 land cover map. The overall Cramer’s V tests show that in the Colombia Amazon region the driving factors that are most closely associated with all classes of LULC were as follow: distance to floodplain forests, distance to Amazon forests, and Amazon forest patch sizes (Cramer’s V ≥ 0.35). Subsequently, other driving factors were useful to explain the process of land transformation: distance to pastures and crops, distance to major rivers, distance to connected agricultural areas, cumulative distance of the agricultural landscape advancement between 2002 and 2016 and physiographic landscape types (Cramer’s V ≥ 0.30). The explanatory variables that obtained the highest values of the Cramer's V global (≥ 0.15) coefficient were chosen. The 29 selected variables and the associated coefficients are in Table S2 (supplementary information).

### Performance of the ANN-MLP model

The number of driving factors varied in each transition model, considering that not all the 29 driving factors influence all land cover change processes. The driving factors for each transition model were selected based on the specific influence of the variable on the coverage transition process and by the scientific literature^46,47^. The driving factors and the precision statistics of each transition model are shown in Table S2 (supplementary information).

In general, the ANN-MLP analysis generated satisfactory precision statistics in all cover transitions, indicating a high predictive capacity of the land use change model. The “accuracy rate” values for the transition models were between 70 and 98.2%, while the “skill measure” values ranged from 0.41 to 0.97, considering in both cases adequate performance results for the transition models of changes in land covers. In the regeneration submodel, the transition from "pastures and crops" to "grasslands and shrubs" with 21 explanatory variables showed the highest AR, with a value of 98.2%, and an SM of 0.97. In the submodel of substitution, the transition from "fragmented forests and secondary vegetation" to "pastures and crops" with 23 explanatory variables, showed the lowest AR with a value of 70.5% and an SM of 0.41. Finally, in the submodel of degradation, the transitions showed AR values above 93% and SM values between 0.86 and 0.95 (Table S2) (supplementary information).

### Validation of the simulation of the changes in the coverage until 2018

The results of the concordance indices to validate the simulated land cover map for 2018 with the SINCHI institute land covermap for 2018, indicated that the coverage prediction model produced by the LCM is useful for projecting scenarios until 2040. The general kappa index, which evaluates the agreement in terms of amount and spatial location of changes in coverage between maps, showed a value of 0.91 that is considered satisfactory, indicating a high degree of agreement between the maps.

The KIA values indicated that the change assignments were better than a chance assignment of the different land covers. KIA results showed that the highest values of concordance between the maps corresponded to the categories of "floodplain forests" and "water bodies and wetlands" (0.99), "grasslands and shrubs" (0.98) and “Amazon forest” (0.97). The lowest concordance was in the vegetation covers "fragmented forest and secondary vegetation" and "pastures and crops", with a KIA of 0.65 and 0.77 (Table 3).

**Table 3:** Validation statistics. Observed map 2018 vs simulated map 2018.

LULC classes	SINCHI 2018 KIA	Predicción 2018 KIA
Floodplain forests	1.00	0.99
Amazon forests	0.97	0.97
Fragmented forests and secondary vegetation	0.60	0.65
Grasslands and shrublands	0.92	0.98
Pastures and crops	0.84	0.77
Urban and artificialized cover	0.89	0.91
Water bodies and wetlands	0.98	0.99
Overall Kappa	0.91	

### Simulation of the scenarios until 2040: from narratives of land use change maps

**Figure 5:**
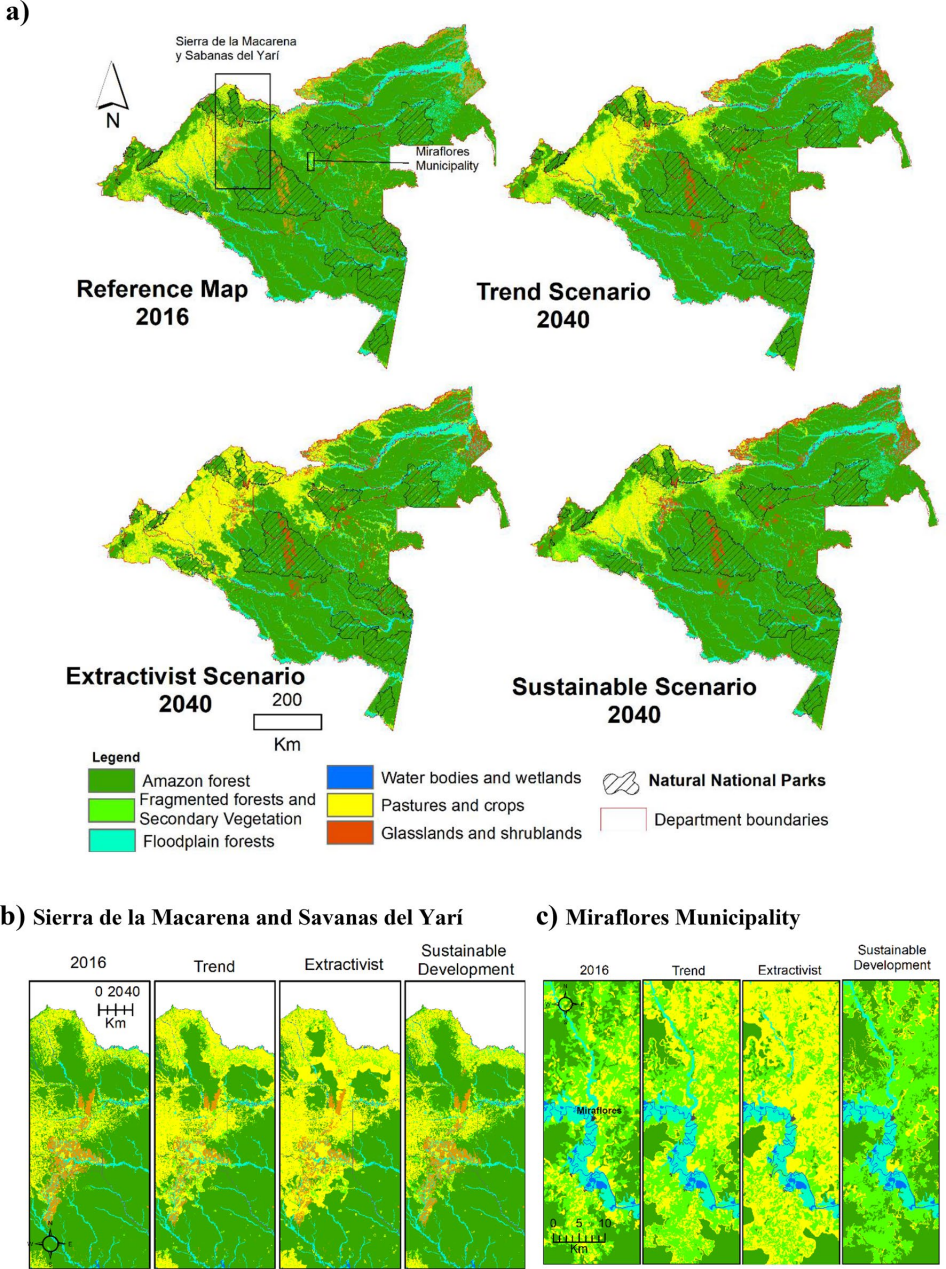
(**a**) Land use and land cover changes under different scenarios from 2016 to 2040. Details of land cover in the different scenarios. (**b**) sector of the Sierra de la Macarena and Tinigua national natural park in the department of Meta, and in the natural savannahs of Yarí in the department of Caquetá. (**c**) Surrounding area of the municipality of Miraflores in the department of Guaviare. (The projection of the LULCC scenarios generated using TerrSet v.19 https://clarklabs.org/terrset/) (Maps generated using software ArcGis 10.7.1 https://www.esri.com).

The land use demands for the coverage in the extractivist and sustainable development scenarios were entered into the LCM through external model matrices that were calculated from the Markov probability matrix of the trend scenario until 2040 (Table 4). The probability matrices included in each scenario are shown in Tables 5 and 6. The coverage projections in each scenario were estimated based on the expectations derived from the elaborated narratives. The spatial parameterization of each scenario was adjusted, which included the main constraints or incentives mentioned in the narratives that acted on the coverage. Subsequently, the spatial distribution of the coverage was predicted until 2040. The results of the simulation of the scenarios are shown in Table 7 and Fig. 5.

**Table 4:** Markovian prediction for 2040 based on LULC cover maps of 2002 and 2016. Scenario BAU.

LULC classes	(FpF)	(AF)	(Ff-Sv)	(Gl-Sl)	(P-C)	(UAC)	(Wb-Wl)
Floodplain forests (FpF)	0.95	0.00	0.01	0.00	0.02	0.00	0.00
Amazon forests (AF)	0.00	0.67	0.12	0.00	0.20	0.00	0.00
Fragmented forests and secondary vegetation (Ff-Sv)	0.01	0.06	0.32	0.00	0.59	0.00	0.00
Grasslands and shrublands (Gl-Sl)	0.00	0.00	0.02	0.84	0.12	0.00	0.00
Pastures and crops (P-C)	0.00	0.00	0.19	0.01	0.78	0.00	0.00
Urban and artificial cover (UAC)	0.00	0.00	0.02	0.04	0.03	0.90	0.00
Water bodies and wetlands (Wb-Wl)	0.00	0.00	0.00	0.00	0.08	0.00	0.90

**Table 5:** Markovian prediction for 2040. External model. Extractivist Scenario.

LULC classes	(FpF)	(AF)	(Ff-Sv)	(Gl-Sl)	(P–C)	(UAC)	(Wb-Wl)
Floodplain forests (FpF)	0.87	0.00	0.01	0.00	0.10	0.00	0.00
Amazon forests (AF)	0.00	0.88	0.02	0.00	0.08	0.00	0.00
Fragmented forests and secondary vegetation (Ff-Sv)	0.01	0.06	0.15	0.84	0.12	0.00	0.00
Pastures and crops (P-C)	0.00	0.00	0.09	0.01	0.88	0.01	0.00
Urban and artificial cover (UAC)	0.00	0.00	0.00	0.00	0.07	0.92	0.00
Water bodies and wetlands (Wb-Wl)	0.00	0.00	0.00	0.00	0.08	0.00	0.90

**Table 6:** Markovian prediction for 2040. External model. Sustainable Development Scenario.

LULC classes	(FpF)	(AF)	(Ff-Sv)	(Gl-Sl)	(P-C)	(UAC)	(Wb-Wl)
Floodplain forests (FpF)	0.95	0.00	0.03	0.00	0.01	0.00	0.00
Amazon forests (AF)	0.00	0.97	0.00	0.00	0.02	0.00	0.00
Fragmented forests and secondary vegetation (Ff-Sv)	0.07	0.17	0.73	0.00	0.01	0.00	0.00
Grasslands and shrublands (Gl-Sl)	0.00	0.00	0.02	0.96	0.01	0.00	0.00
Pastures and crops (P-C)	0.00	0.00	0.27	0.01	0.71	0.00	0.00
Urban and artificial cover (UAC)	0.00	0.00	0.02	0.04	0.03	0.90	0.00
Water bodies and wetlands (Wb-Wl)	0.00	0.00	0.00	0.00	0.08	0.00	0.90

**Table 7:** Area (ha) of LULC classes in the reference year (2016) and under three scenarios until 2040.

			Scenarios 2040					
LULC classes	Reference year 2016	%	BAU	%	Extractivist	%	Sustainable development	%
Floodplain forests	4,288,790	8.9	4,142,512	8.5	3,780,879	7.8	4,210,149	8.7
Amazon forests	35,673,007	73.9	33,779,460	70.0	31,826,314	65.7	35,123,495	72.8
Fragmented forests and secondary vegetation	1,824,387	3.8	2,223,369	4.6	1,773,849	3.6	2,681,416	5.5
Grasslands and shrublands	1,895,873	3.9	1,678,145	3.5	1,653,770	3.4	1,882,283	3.9
Pastures and crops	3,970,030	8.2	5,878,568	12.2	8,617,274	17.8	3,804,739	7.8
Urban and artificial cover	13,380	0.1	13,380	0.1	13,380	0.1	13,380	0.1
Water bodies and wetlands	581,340	1.2	531,372	1.1	581,340	1.2	531,344	1.1

### Patterns of change in future land-use and coverage

As shown in Table 7, the area of “pastures and crops” increased in the trend as well as extractivist scenarios (48% and 117%), while in the sustainable development scenario, the area of "pastures and crops" was stabilized and even decreased by a small percentage (− 4.1%). The most notable dynamics of change occurred in “fragmented forests and secondary vegetation” where, depending on the scenario, this vegetation might be involved in a process of replacement or regeneration. The dynamics of land overuse implied that large areas of "fragmented forests and secondary vegetation" would be converted into "pastures and crops", while a sustainable territorial ordering would conserve "fragmented forests and secondary vegetation" present in 2016 and generated new areas of secondary vegetation in the transformed areas that are the basis for recovery of Amazonian forests by 2040. On the other hand, the trend scenario predicted an increase in "fragmented forests and secondary vegetation" through degradation of intact forests. The connectivity of forest cover with other natural regions was affected in all scenarios, especially towards the Andean region where the loss of forests was notable in the trend and extractivist scenarios. However, in the extractivist scenario, the model identified significant changes that would radically alter the mountain landscape through high loss and forest fragmentation.

### Trend scenario (BAU)

Colonization of forest ecosystems continued in the Colombian Amazon, with ~ 2 million hectares of intact forests lost by 2040. “Pastures and crops” were expanding over forests located on the banks of the Caquetá and Caguán rivers, affecting the municipalities of Solano, Cartagena del Chairá and San Vicente del Caguán. In the municipalities of San José del Guaviare, El Retorno, Calamar and Miraflores in the Department of Guaviare the loss of forests to "pastures and crops" increased, strengthening the deforestation corridor on the banks of the Vaupés River. In the transformed areas of the Department of Caquetá, the landscape was simplified through deforestation of the remaining forest fragments. In the Department of Putumayo, large tracts of intact forests transformed into “fragmented forests and secondary vegetation”. In the Andean-Amazon region, the headwaters of the main rivers that flow into the Amazon plain, the area of "pastures and crops" and "fragmented forests and secondary vegetation" increased, and forest cover in this mountain area was reduced. In the municipality of Santa Rosa, Department of Cauca, deforestation affected the forests at the source of the Caquetá River.

### Extractivist scenario

The spatial pattern of hedging changes was similar to the trend scenario. The extent of Amazon forests (amazon forests and floodplain forests) was reduced to 73.5% (~ 35.6 million hectares), with a loss of ~ 4.3 million hectares. Crops and pastures increased 117%, from ~ 3.97 million hectares in 2016 to ~ 8.61 million hectares in 2040. The agricultural landscape was further simplified by the replacement of “fragmented forests and secondary vegetation” by "pastures and crops". In this scenario, the advance of deforestation near the Caquetá, Caguán and Putumayo rivers would cause large blocks of continuous forests to separate and become isolated. In the Andean-Amazon region, there was a breakdown of large extensions of forests along the mountain range. This caused the Cordillera de los Picachos, Tinigua and Sierra de la Macarena National Natural Parks to are divided, and one of the most important areas of structural connectivity between Amazonian and Andean forests and of these two ecosystems with the Orinoquía natural savannas, was interrupted (Fig. 5b). Deforestation was consolidating along the Vaupés river, increasing “pastures and crops” around the municipality of Miraflores in the Department of Guaviare (Fig. 5c) and in the municipality of Mitú in the Department of Vaupés. The advancement of "pastures and crops" that enter the Orinoquía region led to the loss of large extensions of "grasslands and shrubs" with a natural origin, characteristic coverage in the Orinoquía-Amazonia transition.

### Sustainable development scenario

In this scenario, 2.1% (~ 0.87 million hectares) of the forests were lost between 2016 and 2040. There was a significant reduction in the transformation of forests to "pastures and crops" in the departments Caquetá, Meta, Guaviare and Putumayo, which increased the permanence of a large part of Amazonian forests by 2040. Proper management of the transformed areas encouraged the permanence of “fragmented forests and secondary vegetation” in the departments Putumayo and Guaviare, as well as along the Andean mountain range. These recovering forests compensated for deforestation events. Around populated centers, areas of “pastures and crops” were enriched with a dominant matrix of “fragmented forests and secondary vegetation”, especially in Florencia-Caquetá; Miraflores- Guaviare (Fig. 5b); and Mitú-Vaupés, where productive restoration programs and silvopastoral systems were implemented. Around the Sierra de la Macarena National Park, the landscape was simplified by deforestation of small fragments of forests; however, within the PA, the integrity of forests was maintained, guaranteeing the Andes-Amazonia structural connection.

## Discussion

In this research, land use and land cover changes were modeled using ANN-MLP combined with a Markov chain model and the design of a set of story lines for the future, effectively simulating three scenarios of land-use change by 2040 for the Colombian Amazon: trend, extractive and sustainable development. The results could be used by the Colombian government and environmental authorities as a scientific tool to guide decision-making on deforestation control, to identify areas where the expansion of pastures and crops could occur, and to implement actions at the limits of the protected areas to avoid deforestation. Likewise, the sustainable development scenario showed that the implementation of sustainable productive restoration practices in transformed lands and the protection of vegetation, would provide secondary fragmented forests and increase these covers that are key to providing a carbon sink to reduce the climate change effects, biodiversity recovery and ecosystem services^62–65^.

Results showed that the distance to floodplain forests and Amazonian forests, as well as the size of forest patches, were important drivers of the process of land cover change, indicating that the greatest threat to Amazonian forests was the presence of forests per se. It is counter-intuitive to this statement, but the results indicated that while the distance of Amazonian forests from pastures and crops was important, it is not the main driving factor. There was a systematic behavior of logging and the advance of the agricultural frontier brought the actors of deforestation closer to new forests rather than generating productive activities on the newly available areas due to the intentional loss of forests producing unsustainable land grabbing^4^.

The narrative construction technique was notable, because it was useful for understanding how socio-political and economic factors, ancestral human populations (indigenous peoples), and historical and recent colonization processes would determine the future of forests and natural ecosystems in the Colombian Amazon. A key factor in the narratives was the fulfillment or not of the peace agreement and the commitments for comprehensive rural reform and substitution of illicit coca crops. These decisions could mark paths for the construction of territorial development and point towards an optimistic or pessimistic scenario in the current post-conflict stage. The narratives and land cover maps simulated up to 2040 made this research an adequate tool for territorial planning at different scales for the Colombian Amazon in the near future.

The high resolution of the scenarios (60 m pixel) made them useful at the municipal level, where they are key to discussing the future of the region in land-use planning plans. Other scenarios for the Colombian Amazon that address the future impact of agricultural policies (pasture expansion) on intact forests with a pixel resolution of 100 m^8^, showed that forest losses may be even greater by 2030 (~ 3 million hectares) than reported in this research until 2040 (~ 2 million hectares). However, in this research, the extractivist scenario showed that the loss of forests can reach ~ 4.3 million hectares by 2040. This implies a higher demand for land than expected in the trend scenario because changes in global consumption patterns could increase the environmental degradation of the Amazon, increasing social conflicts and economic inequality in the region.

The approach to modeling changes in covers obtained high precision in the analyzed transition submodels. However, in the LCM, it was not possible to visualize the behavior of the explanatory variables in each transformational process. Therefore, to improve an understanding of the factors that influence changes in covers, transitions should be analyzed with another method that analyzes non-linear relationships and obtains the visual response and contribution of the variable in each submodel.

## Conclusion

Based on the analysis of the transitions for change in the coverage between 2002 and 2016 using ANN-MLP combined with the Markov model and using a set of plausible narratives for the future, a set of spatially explicit scenarios for land-use cover in the Colombian Amazon until 2040 was created. The scenarios and simulations developed in this research are strategic in nature and, as such, they provide a general guide for the aspects of coverage "on average” under different management policies. The characteristics of the narratives and changes in land cover under each scenario for 2040 are as follow:

1. It was possible to consolidate a reduced set of contrasting scenarios that allowed understanding the consequences of different alternatives in the future of forests in the Colombian Amazon. The knowledge of experts on driving forces that transform the Amazon made the scenarios plausible with a high level of credibility.
2. Under the three scenarios, the resulting simulations revealed the strong effect of deforestation in the foothills, Sierra de la Macarena, and the disconnection with the Andean forests in the trend scenario, as in the extractivist scenario. Similarly, in addition to the deforestation arc in Caquetá, the deforestation fronts would expand in Guaviare, Meta, Vichada and Putumayo. Similarly, in the Vaupés region, near Mitú, the deforestation border could expand, which already has a history of doing so.
3. Under the sustainable development scenario, “fragmented forests and secondary vegetation” are of great importance because management decisions aimed at increasing their permanence favor the natural restoration of advanced successional forests. Similarly, conservation actions will promote secondary vegetation matrices in areas of restoration, improving friction surfaces in transformed areas and encouraging structural and functional connectivity in intervened areas. Preserving the intact forests that currently cover the Colombian Amazon is, in all cases, the best conservation scenario.

## Supplementary Information

Supplementary Tables.

## Data Availability

The authors declare compliance with Scientific Reports’ policy regarding the data availability. All relevant data are within the paper and its Supplementary Information files, and any other information about this paper can be given upon request to the corresponding author.
