# Supplementary material for: Scenarios of land use and land cover change in the Colombian Amazon to evaluate alternative post-conflict pathways
Source: Sci Rep. 2023 Feb 7;13:2152. doi: 10.1038/s41598-023-29243-2 (PMC9905563; doi:10.1038/s41598-023-29243-2)
Supplement: Supplementary file 1 — Supplementary Tables. [file 41598_2023_29243_MOESM1_ESM.docx]

**SUPLEMENTARY INFORMATION**

Scenarios of land use and land cover change in the Colombian Amazon to evaluate alternative post-conflict pathways

**AUTHORS**

Agudelo-Hz, William-J^1^*; Castillo-Barrera, Natalia-C^1^ & Murcia-García Uriel^1^

**AFFILIATION**

^1^GIS and RS Laboratory, Functioning Models and Sustainability Program, Amazon Institute for Scientific Research SINCHI, Bogotá, Colombia, Calle 20 # 5-44

Table S1. Variables tested in land use land cover change model. This table show the source and description of each driving factor considered.

| Theme | Driving factor | Source | Description |
| --- | --- | --- | --- |
| Accessibility | 1. Density of roads/tracks | Agustín Codazzi Geographic Institute | Ratio between the length of the total road network (m) and a unit area (km^2^) |
|  | 2. Distance to towns | Agustín Codazzi Geographic Institute | Euclidean distance to the towns (m) |
|  | 3. Distance to major rivers | Agustín Codazzi Geographic Institute | Euclidean distance to major rivers (m) |
|  | 4. Distance to secondary rivers | Agustín Codazzi Geographic Institute | Euclidean distance to secondary rivers (m) |
|  | 5. Distance to roads/tracks | Agustín Codazzi Geographic Institute | Euclidean distance to roads/tracks (m) |
| Topographics | 6. Aspect – eastness | DEM - ASTER V003 | Provide continuous measures (-1 to +1), describing the orientation of the slopes |
|  | 7. Aspect – northness | DEM - ASTER V003 |  |
| Climate | 8. BIO4 | Worldclim 2.0 | Temperature Seasonality (°C) |
|  | 9. BIO9 | Worldclim 2.0 | Mean Temperature of Driest Quarter (°C) |
|  | 10. BIO12 | Worldclim 2.0 | Annual Precipitation (mm) |
| Landscape features | 11. Patch sizes fragmented forests | SIMCOBA – SINCHI Institute | Ranges of patch sizes of fragmented forests (ha) |
|  | 12. Patch sizes floodplain forests | SIMCOBA – SINCHI Institute | Ranges of patch sizes of floodplain forests (ha) |
|  | 13. Patch sizes amazon forests | SIMCOBA – SINCHI Institute | Ranges of patch sizes of amazon forests (ha) |
|  | 14. Patch sizes grasslands and shrublands | SIMCOBA – SINCHI Institute | Ranges of patch sizes of grasslands and shrublands |
|  | 15. Distance to grasslands and shrublands | SIMCOBA – SINCHI Institute | Euclidean distance to grasslands and shrublands (ha) |
|  | 16. Distance to fragmented forests | SIMCOBA – SINCHI Institute | Euclidean distance to fragmented forests (m) |
|  | 17. Distance to floodplain forests | SIMCOBA – SINCHI Institute | Euclidean distance to floodplain forests (m) |
|  | 18. Distance to amazon forests | SIMCOBA – SINCHI Institute | Euclidean distance to amazon forests (m) |
| Production practices and environmental degradation | 19. Livestock density | Colombian Agricultural Institute -ICA- | Ratio between the number of animals (livestock) and a unit area (km^2^). |
|  | 20. Fire density (hot spots) | SIATAC - SINCHI Institute | Ratio between the number of hot spots in a unit of surface area (km^2^) |
|  | 21. Distance to illicit crops (coca) | SIMCI-UNODC | Euclidean distance to coca field found between 2002 and 2008 (m) |
|  | 22. Distance to pastures and crops | SIATAC - SINCHI Institute | Euclidean distance to pastures and crops (m) |
|  | 23. Distance to isolated agricultural areas (enclaves) | SIATAC - SINCHI Institute | Euclidean distance to small agricultural areas generally located inside forests (e.g., chagras) (m) |
|  | 24. Distance of the agricultural landscape advancement | SIATAC - SINCHI Institute | Cumulative distance of the agricultural landscape between 2002 and 2016 (m) |
|  | 25. Distance to connected agricultural areas | SIATAC - SINCHI Institute | Euclidean distance to agricultural areas connected by road and tracks to the major urban centers in the interior of the country (m) |
|  | 26. Distance to areas with mining titles | National Mining Agency | Euclidean distance to areas licensed by the state for mineral extraction (m) |
| Socioeconomic | 27. Population density | DANE | Number of people per unit of area (per km^2^) |
|  | 28. Gross domestic product 2002 | DANE | Total monetary or market value of all the finished goods and services produced in the Amazon region in 2002 |
|  | 29. Gross domestic product 2016 | DANE | Total monetary or market value of all the finished goods and services produced in the Amazon region in 2016 |
|  | 30. Difference in Gross Domestic Product between 2002 and 2016 | DANE | Difference in the Total monetary or market value of all the finished goods and services produced in the Amazon region between 2002 and 2016 |
|  | 31. GINI Index 2005 | DANE | Measure of the distribution of income in the amazon population in 2005 |
|  | 32. GINI Index 2014 | DANE | Measure of the distribution of income in the amazon population in 2014 |
|  | 33. Difference in GINI 2005 and 2014 | DANE | Difference in the measure of the distribution of income in the amazon population between 2005 and 2014 |
|  | 34. Legal status of territory | SIMCOBA – SINCHI Institute | Legal status of the territory based on the Land Management Plans |
|  | 35. Distance to indigenous settlements | Ministry of the Interior | Euclidean distance to indigenous settlements (m) |
| Landscape management | 36. Distance to National Natural Parks | National System of Protected Areas (SINAP) | Euclidean distance to National Natural Parks (m) |
|  | 37. Distance to Indigenous Reservations | Ministry of the Interior | Euclidean distance to Indigenous Reservations (m) |
|  | 38. Distance to the subtraction areas of the forest reserve (Law 2nd) | Ministry of Environment and  Sustainable Development | Euclidean distance to subtraction areas of the forest reserve (m) |
|  | 39. Distance to forest reserve areas in the Amazon (Law 2nd) | Ministry of Environment and Sustainable Development | Euclidean distance to forest reserve areas in the Amazon (Law 2nd) (m) |
| Soil characteristics | 40. Soil drainage quality | Agustín Codazzi Geographic Institute | Map with different categories of drainage quality of Amazonian soils |
|  | 41. Physiographic landscape types | Agustín Codazzi Geographic Institute | Map with different categories of physiographic landscape types |

Table S2. Globlal Cramer's V values for the selected variables and precision statistics for the land use/land cover transition sub-models and the respective variables used in each model. Floodplain Forests (FPF), Grassland and Shrubland (GLSL), Amazon forets (AF), Forests fragmented and Secondary vegetation (FFSV), Pastures and crops (PC), Water bodies and wetlands (WBWL).

|  | | **DEGRADATION** | | | | **SUBSTITUTION** | | | | | **REGENERATION** | | | | |
| --- | --- | --- | --- | --- | --- | --- | --- | --- | --- | --- | --- | --- | --- | --- | --- |
| **Driving factors** | **Global Cramer's V** | **FPF to GLSL** | **FPF to FFSV** | **AF to GLSL** | **AF to FFSV** | **GLSL to PC** | **WBWL to PC** | **FPF to PC** | **FFSV to PC** | **AF to PC** | **PC to FFSV** | **GLSL to FFSV** | **PC to GLSL** | **FFSV to FPF** | **FFSV to AF** |
| 1. Patch sizes Fragmented forests | 0.19 |  |  |  |  |  |  |  | X |  |  |  |  |  | X |
| 2. Patch sizes Amazon Forests | **0.35** |  |  | X |  |  |  |  |  | X |  |  |  |  |  |
| 3. BIO 9 | 0.17 | X | X | X |  |  | X | X | X |  | X |  | X | X | X |
| 4. Livestock density | 0.16 |  | X | X | X | X |  |  | X |  | X |  |  |  |  |
| 5. Fire density (hot spots) | 0.20 |  | X |  | X |  |  | X | X | X | X | X |  | X |  |
| 6. Population density | 0.18 |  | X | X | X | X | X | X | X |  | X | X | X | X | X |
| 7. Density of roads/tracks | 0.24 |  |  |  | X |  |  |  | X | X |  | X |  | X |  |
| 8. Distance to Grasslands and Shrublands | 0.37 | X |  | X |  |  | X |  |  |  | X |  | X | X |  |
| 9. Distance to fragmented forests | 0.20 | X | X | X |  | X | X | X |  |  | X |  | X |  |  |
| 10. Distance to floodplain forests | **0.38** |  |  |  |  |  | X |  |  |  |  |  |  | X |  |
| 11. Distance to amazon forests | **0.36** |  |  |  |  | X |  | X | X |  | X | X | X |  | X |
| 12. Distance to towns | 0.28 | X |  |  | X |  | X | X | X |  | X | X |  | X | X |
| 13. Distance to illicit crops (coca) | 0.24 | X | X | X | X |  | X | X |  | X | X | X | X | X | X |
| 14. Distance to pastures and crops | **0.31** | X | X | X |  |  | X |  | X | X |  | X |  | X | X |
| 15. Distance to major rivers | **0.31** | X | X | X | X |  | X | X | X | X | X | X | X | X | X |
| 16. Distance to connected agricultural areas | **0.31** | X | X | X | X | X | X | X | X | X | X | X | X | X | X |
| 17. Distance to National Natural Parks | 0.16 | X | X | X | X | X | X |  | X | X | X | X | X | X | X |
| 18. Distance to Indigenous Reservations | 0.15 | X | X | X |  | X | X | X | X | X | X | X | X | X | X |
| 19. Distance to the subtraction areas of the forest reserve (Law 2nd) | 0.19 | X | X | X | X | X | X | X | X | X | X | X | X | X | X |
| 20. Cumulative distance of the agricultural landscape advancement (2002-2016) | **0.30** | X | X |  | X | X | X | X | X | X | X | X | X | X | X |
| 21. Distance to isolated agricultural areas (enclaves) | 0.22 |  | X |  | X | X | X | X | X | X | X | X | X | X | X |
| 22. Distance to areas with mining titles | 0.15 |  |  | X | X | X | X | X | X | X | X |  | X | X | X |
| 23. Distance to roads/tracks | 0.27 | X | X | X | X | X | X | X | X | X | X | X | X | X | X |
| 24. Legal status of territory | 0.24 | X | X | X | X |  | X |  | X | X | X | X | X |  | X |
| 25. Gross domestic product 2002 | 0.17 | X | X | X | X | X |  | X | X |  | X | X | X | X | X |
| 26. Gross domestic product 2016 | 0.17 | X | X | X |  | X | X | X | X |  | X | X | X | X | X |
| 27. Difference in GDP between 2002-2016 | 0.17 | X | X | X | X |  | X | X | X |  | X | X | X | X | X |
| 28. Soil drainage quality | 0.24 | X |  | X | X | X |  | X | X |  |  | X | X | X | X |
| 29. Physiographic landscape types | **0.31** | X | X | X | X | X | X | X |  |  |  | X | X | X | X |
| **Number of variables** | | 19 | 20 | 21 | 19 | 16 | 21 | 20 | 23 | 15 | 22 | 21 | 21 | 23 | 22 |
| **Model precision statisticals** | | Accuracy rate | Accuracy rate | Accuracy rate | Accuracy rate | Accuracy rate | Accuracy rate | Accuracy rate | Accuracy rate | Accuracy rate | Accuracy rate | Accuracy rate | Accuracy rate | Accuracy rate | Accuracy rate |
|  |  | 93.10 | 95.94 | 96.66 | 97.42 | 95.35 | 92.90 | 95.92 | 70.50 | 97.63 | 71.07 | 97.70 | 98.27 | 88.64 | 71.05 |
|  |  | Skill measure | Skill measure | Skill measure | Skill measure | Skill measure | Skill measure | Skill measure | Skill measure | Skill measure | Skill measure | Skill measure | Skill measure | Skill measure | Skill measure |
|  |  | 0.86 | 0.92 | 0.93 | 0.95 | 0.91 | 0.86 | 0.92 | 0.41 | 0.95 | 0.42 | 0.95 | 0.97 | 0.77 | 0.42 |
